# IMPLEMENTATION MODELS FOR FOLLOWING UP AFTER A DEMENTIA-RELATED MISSING INCIDENT

**DOI:** 10.1093/geroni/igad104.0282

**Published:** 2023-12-21

**Authors:** Christine Daum, Elyse Letts, Lauren McLennan, Cathy Conway, Lili Liu

**Affiliations:** University of Waterloo, Waterloo, Ontario, Canada; University of Waterloo, Waterloo, Ontario, Canada; University of Waterloo, Waterloo, Ontario, Canada; University of Waterloo, Waterloo, Ontario, Canada; University of Waterloo, Waterloo, Ontario, Canada

## Abstract

The rate of persons living with dementia who go missing is a growing concern, especially those who go missing repeatedly. Drawing upon the practices of other populations such as at-risk youth, following up with persons living with dementia and their supports after an incident could reveal contributing factors and also connect people with supports to mitigate the risk for going missing again. In Canada, models for such follow-ups are limited. The purpose of this presentation is to describe two implementation models for following up with persons living with dementia and their care partners after a missing incident has occurred. We conducted 20 individual semi-structured online interviews with police and service providers in Canada and the United Kingdom to understand the scope of and approaches to following up with this population. Generic qualitative description and conventional content analysis were used. We developed a resource guide that describes these approaches and proposes how follow-ups could be consistently employed. Two focus groups with police officers and community service providers (n= 11) were conducted to obtain feedback on the guide. We are now working with community partners in two Canadian provinces to develop implementation models unique to their existing processes, resources, and systems; these models put into practice approaches in the resource guide. Our presentation will showcase the guide and the models developed in collaboration with our partners. These models can guide police organizations, Alzheimer Societies, and communities who support persons living with dementia at risk of going missing.

